# Pretransplant Nephrectomy for Large Polycystic Kidneys in ADPKD (Autosomal Dominant Polycystic Kidney Disease) Patients: Is Peritoneal Dialysis Recovery Possible after Surgery?

**DOI:** 10.1155/2019/7343182

**Published:** 2019-03-25

**Authors:** Giuseppe Ietto, Veronica Raveglia, Elia Zani, Domenico Iovino, Cristiano Parise, Gabriele Soldini, Nicholas Walter Delfrate, Lorenzo Latham, Giovanni Saredi, Fabio Benedetti, Matteo Tozzi, Giulio Carcano

**Affiliations:** ^1^General, Emergency and Transplant Surgery Department, Ospedale di Circolo e Fondazione Macchi, University of Insubria, Varese, Italy; ^2^Urology Department, Ospedale di Circolo e Fondazione Macchi, Varese, Italy

## Abstract

The choice of modality for renal replacement therapy in patients with ADPKD varies, often based on patient choice, physician-related factors, and resource availability. For a long time peritoneal dialysis (PD) was considered as relative contraindication due to the possible limited intraperitoneal space. In recent years, some studies suggested it is a valid option also in patients with ADPKD to be considered as a first line treatment in potentially fit patients. Diuresis volume lowering and potential permanent damage of peritoneal integrity, both leading to a necessary switch to haemodialysis, are the two most important dangers after nephrectomy, especially if bilateral, in PD patients. We performed a retrospective analysis of patient underwent native polycystic kidney nephrectomy in order to state the possibility to recover peritoneal dialysis after surgery.

## 1. Introduction

**Figure 1:**
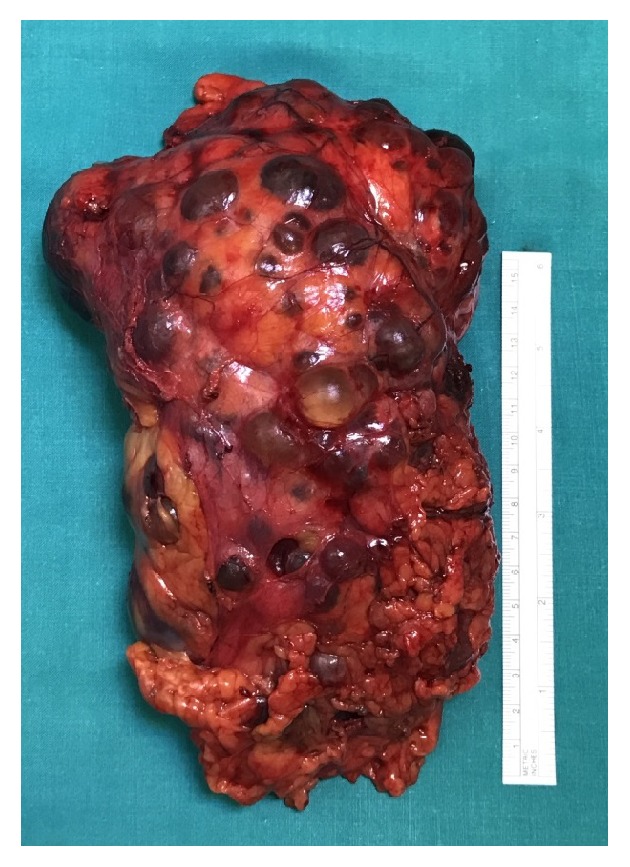
Renal parenchima is largely replaced by cysts that may become haemorrhagic or infected.

The term polycystic kidney disease should be reserved for two hereditary diseases: autosomal recessive polycystic kidney disease (ADPKD) and, most commonly, autosomal dominant polycystic kidney disease (ADPKD) [1, 2]. ADPKD is characterized by relentless development and growth of cysts causing progressive kidney enlargement associated with hypertension, abdominal fullness and pain, episodes of cyst haemorrhage giving rise to gross haematuria, nephrolithiasis, cyst infections, and progressive renal failure (Figure 1) [3]. ADPKD is one of the most common human hereditary diseases and leads to end stage renal disease requiring dialysis or renal transplantation in about 50% of the patients [4, 5]. Diuresis and fluid volume control is often preserved also during the dialytic period [6, 7].

**Figure 2:**
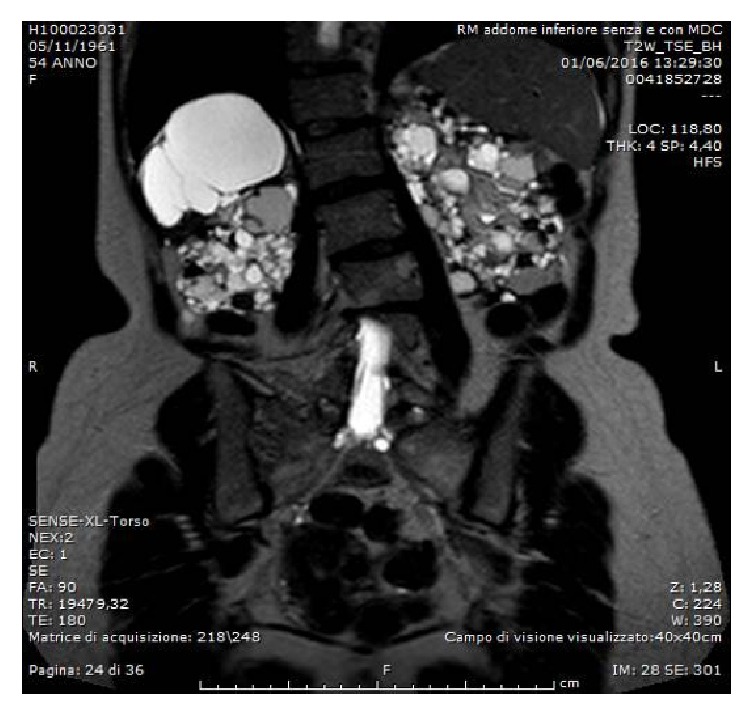
Preoperative MR scan shows size of the polycystic kidneys and eventually haemorrhagic or infected cysts and suspicious areas of malignancy [18].

Before transplantation native kidneys are not routinely removed considering significant morbidity and mortality associated [8, 9]. Indications for nephrectomy include recurrent and/or severe infection, symptomatic nephrolithiasis, recurrent and/or severe bleeding, intractable pain, suspicion of renal cancer, and space restrictions prior to transplantation (Figure 2), taking into account that kidney size typically declines after transplantation [10–13].

In recent years, some studies suggested peritoneal dialysis (PD) as valid option also in patients with ADPKD [14], which was considered a relative contraindication previously due to the possible limited intraperitoneal space to accommodate the dialytic fluid, as well as the risk of hernia [15]. The optimum time for native polycystic kidney nephrectomy is still a topic of debate in the scientific literature. Many factors have to be considered to make the right choice for each patient: dialysis or pre-emptive transplantation, complication severity, anuria, easy access to transplantation, and potential living donor. For those patients waiting for transplantation, requiring renal replacement therapy, either haemodialysis (HD) or peritoneal dialysis (PD) is suitable modalities. The choice of modality for renal replacement therapy in patients with ADPKD varies, often based on patient choice, physician-related factors, and resource availability. Although intra-abdominal space restrictions, increased risk for abdominal wall hernias, and increased prevalence of colonic diverticula may pose challenges, ADPKD is not a contraindication for PD. For patients whom started PD as replacement therapy native polycystic nephrectomy may be harmful considering the manipulation and possible damage of peritoneum required by the procedure. After surgery, it may be no more efficient in supporting peritoneal dialysis. Therefore, the choice for nephrectomy is more difficult for ADPKD patient in PD.

We performed a retrospective analysis of patient underwent native polycystic kidney nephrectomy in order to state the possibility to recover peritoneal dialysis after surgery.

## 2. Materials and Methods

### 2.1. Patients and Surgical Technique

A retrospective analysis of ADPKD patients in waiting list for kidney transplant, which underwent nephrectomy in our transplant surgery department between December 2012 and December 2017, was carried out. All patients were included, male and female, and the indications for nephrectomy were symptoms (pain, urinary tract infections, or hematuria) or kidney size responsible of subocclusive symptoms or difficulty for future graft positioning.

In all cases, nephrectomy was performed with laparotomic transperitoneal surgical technique through subcostal incision. The whole procedure was carried out using the harmonic scalpel Ultracision® (Ethicon US, LLC) in order to limit postsurgical bleeding and lymph spread. After completing nephrectomy, the posterior peritoneal flap was always reconstructed to preserve peritoneal cavity avoiding visceral adhesions for future peritoneal dialysis recovery.

### 2.2. Data Collection

Clinical data, including demographic parameters, biochemical data, and copathology during the surgery, were collected from our database based on a review of patient medical notes. Demographic including the gender, age, cause of ESRD, haemodialysis (HD) or peritoneal dialysis (PD), and comorbid conditions. We considered pre and post-surgery residual diuresis, pre and post nephrectomy creatinine value, the time from the beginning of dialysis to nephrectomy. Indications for nephrectomy include recurrent and/or severe infection, symptomatic nephrolithiasis, recurrent and/or severe bleeding, intractable pain, suspicion of renal cancer, and space restrictions prior to transplantation. The data concerning the intervention include side of procedure, operator surgeon, and any peri- and postoperative complications. The renal volume was defined as the total volume of both kidneys and calculated after the nephrectomy on the specimen. After surgery we considered if residual diuresis was present or not and if peritoneal dialysis was resumed.

## 3. Results

During the last five years thirty-three patients, all affected by ADPKD related ESRD and underwent nephrectomy in our transplant surgery department from December 2012 to December 2017. Among the patients 20 (60%) were males and 13 (40%) were females, with a mean age of 54 ± 8.

Thirty patient were in waiting list for kidney transplant from cadaveric donor (30/33), two of them also were scheduled for living donor transplantation as signaled previously, and three patients were already transplanted (3/33).

The nephrectomy was bilateral in 2 patients (2/33) (6%), and unilateral in 31 (31/33) patients (94%) (20 right - 64% in 11 left -36%). The mean surgical specimen weight was 1975.58 Kg.

Nine patients (9/30) (27.3%) underwent peritoneal dialysis as replacement therapy, twenty-two patients (22/30) (66.7%) underwent hemodialysis, and two (2/30) (6%) patients had a residual renal function yet useful to avoid replacement therapy and were scheduled for living donor transplantation: pre-emptive transplantation (Table 1). Three of nine patients (3.3%) in PD underwent the nephrectomy after the kidney transplant; the indications for the surgery were in all three cases recurrent urinary tract infections.

Simultaneous the nephrectomy two patients underwent the cholecystectomy for cholelithiasis, two underwent prophylactic appendectomy, and one repaired umbilical hernia and two inguinal hernia.

Nephrectomy became necessary in 19/33 (57.7%) of patients considering their symptoms. Among them 52,9% had recurrent UTIs and 11.7% chronic pain and persistent hematuria was the symptom at admission in 35.4% of cases. Quite half of our patients required nephrectomy considering kidney size to improve renal graft positioning avoiding compression (Table 2).

Considered only pretransplanted patients (30/33) the mean value of pre nephrectomy residual dieresis was 1540 ml/die (min 0 ml-max 3500 ml) while, after nephrectomy, the residual diuresis value decreased to 860 ml/die (min 0 ml-max 1500 ml). Prenephrectomy mean creatinine value was 7.86 mg/dl (min 3.14 mg/dl-max 12.78 mg/dl) and postnephrectomy this value increase 8.74 mg/dl (min 1.23 mg/dl- max 13.84 mg/dl). The mean waiting time from begin of dialysis and native nephrectomy was 22.78 months (min 1 month-max 60 months).

All six PD patients underwent positioning a central line during surgery to allow a bridge period of hemodialysis as replacement therapy after nephrectomy in order to obtain a complete healing of peritoneal surgical incisions to avoid extra-peritoneal spread of dialytic solution and to reduce the risk of incisional hernia.

Two patients never recovered peritoneal dialysis after the nephrectomy because of perioperative complications occurred.

Four of six patients started again peritoneal dialysis after a mean time of 35±5 days after surgery. All had preservation of a sufficient diuresis and no difficulty in loading dialytic fluid into the peritoneum or to evacuate it after dialysis. Purifying efficacy of the treatment was satisfactory. No incisional hernia was reported (Table 3).

## 4. Discussion

In many Western countries, peritoneal dialysis (PD) is not often the treatment of choice for ADPKD patients because of the possible limited intraperitoneal space to accommodate the dialysis fluid, as well as the risk of hernia [15]. Recently some studies suggested that PD may be a valid option for dialysis in ADPKD related ESRD [16].

Peritoneal dialysis ensures a better survival during the first two years of therapy and should be therefore employed as a first line treatment in potentially fit patients [17].

Polycystic Kidneys should not be routinely removed prior to transplantation since nephrectomy in ADPKD patients is associated with significant morbidity and mortality. Indications for nephrectomy include recurrent and/or severe infection, symptomatic nephrolithiasis, recurrent and/or severe bleeding, intractable pain, suspicion of renal cancer, and space restrictions prior to transplantation (even considering that kidney size typically declines after transplantation) [18]. Brazda et al. [19, 20] in their series reported a higher rate of native nephrectomy (35.4%) and advocated that if native nephrectomy were needed, it would be better to perform it before transplantation rather than after. In our experience only three patients underwent nephrectomy after kidney transplant for recurrent and worsening UTIs, apparently related to the immunosuppressive regimen.

Nephrectomy in PD patients, especially if bilateral, carries a twofold risk: a decline of diuresis volume and a potential permanent damage of peritoneal integrity, both leading to a necessary switch to hemodialysis. Data retrieved from our series shows that 66.6% of patients who underwent monolateral native nephrectomy prior to transplantation successfully recovered PD within 35 ± 5 days after the surgical operation avoiding, thus, the problems linked to the hemodialytic routine: a worse quality of life and a progressive vascular impairment potentially harmful in view of future transplantation. Differently from what is reported in Hsu et al. [21] case series which demonstrates that concerning the PD recovering, nephrectomy by retroperitoneal route is strongly recommend, in our population it seems that even a transperitoneal approach for the nephrectomy may be a feasible technique and does not interfere with the peritoneal dialysis recovery.

In our experience only those patients who presented postoperative complications, both haemorrhagic, requiring in one of the two cases reintervention, and open abdomen treatment, did not recovered their PD routine.

Obviously there are many limitations in our analysis in particular those linked to the small sample size which do not allows a clear identification of clinically meaningful differences between groups. Furthermore, because of the retrospective design, we lack data concerning liver size or other surrogate measures for internal organ enlargement (e.g., waist circumference). As a result, it is not possible to evaluate correctly the correlation between kidney volume and the clinical outcome (especially the risk of hernia).

## 5. Conclusions

Native polycystic kidney nephrectomy is still a topic of debate. The optimum time is not well established especially for patients undergoing PD as replacement therapy considering the risk of peritoneal damage and HD as the only one possible choice before transplant.

Our experience suggests monolatheral nephrectomy with laparotomic approach as a safe and feasible procedure also for very large kidneys. Performance before transplant with meticulous surgical technique allows peritoneal preservation avoiding common complication and limiting adherence syndrome in order to rapid peritoneal dialysis recovery.

## Figures and Tables

**Table 1:** 

Dialysis	Number of patients
HD	22 (73.3%)
PD	6 (20%)
Pre-emptive transplantation	2 (6.6%)

**Table 2:** 

Indications for nephrectomy	Percentage
Kidney volume	42.3%
Symptoms	57.7%
(i) Urinary Tract Infections (UTIs) recurrence	52.9%
(ii) Persistent hematuria	35.3%
(iii) Chronic pain	11.7%

**Table 3:** 

Dialysis after nephrectomy in PD patients	Percentage
PD patients	6
(i) HD beginning	33.3% - 2 patients
(ii) PD recovery after surgery	66.6% - 4 patients

## Data Availability

The data used to support the findings of this study are available from the corresponding author upon request.
